# Injection Drug Use Frequency Before and After Take-Home Naloxone Training

**DOI:** 10.1001/jamanetworkopen.2023.27319

**Published:** 2023-08-04

**Authors:** Samantha Colledge-Frisby, Kasun Rathnayake, Suzanne Nielsen, Mark Stoove, Lisa Maher, Paul A. Agius, Peter Higgs, Paul Dietze

**Affiliations:** 1 National Drug Research Institute, Curtin University, Melbourne, Victoria, Australia; 2 Disease Elimination Program, Burnet Institute, Melbourne, Victoria, Australia; 3 National Drug and Alcohol Research Centre, University of New South Wales (UNSW) Sydney, Sydney, New South Wales, Australia; 4 Monash Addiction Research Centre, Monash University, Melbourne, Victoria, Australia; 5 The Kirby Institute, UNSW Sydney, Sydney, New South Wales, Australia; 6 Faculty of Health, Deakin University, Melbourne, Victoria, Australia; 7 Department of Public Health, La Trobe University, Melbourne, Victoria, Australia

## Abstract

**Importance:**

Concerns that take-home naloxone (THN) training may lead to riskier drug use (as a form of overdose risk compensation) remain a substantial barrier to training implementation. However, there was limited good-quality evidence in a systematic review of the association between THN access and subsequent risk compensation behaviors.

**Objective:**

To assess whether THN training is associated with changes in overdose risk behaviors, indexed through injecting frequency, in a cohort of people who inject drugs.

**Design, Setting, and Participants:**

This cohort study used prospectively collected self-reported behavioral data before and after THN training of participants in The Melbourne Injecting Drug User Cohort Study (SuperMIX). Annual interviews were conducted in and around Melbourne, Victoria, Australia, from 2008 to 2021. SuperMIX participants were adults who regularly injected heroin or methamphetamine in the 6 months preceding their baseline interview. The current study included only people who inject drugs who reported THN training and had participated in at least 1 interview before THN training.

**Exposure:**

In 2017, the SuperMIX baseline or follow-up survey began asking participants if and when they had received THN training. The first THN training date that was recorded was included as the exposure variable. Subsequent participant interviews were excluded from analysis.

**Main Outcomes and Measures:**

Injecting frequency was the primary outcome and was used as an indicator of overdose risk. Secondary outcomes were opioid injecting frequency, benzodiazepine use frequency, and the proportion of the time drugs were used alone. Fixed-effects generalized linear (Poisson) multilevel modeling was used to estimate the association between THN training and the primary and secondary outcomes. Time-varying covariates included housing status, income, time in study, recent opioid overdose, recent drug treatment, and needle and syringe coverage. Findings were expressed as incidence rate ratios (IRRs) with 95% CIs.

**Results:**

There were 1328 participants (mean [SD] age, 32.4 [9.0] years; 893 men [67.2%]) who completed a baseline interview in the SuperMIX cohort, and 965 participants completed either a baseline or follow-up interview in or after 2017. Of these 965 participants, 390 (40.4%) reported THN training. A total of 189 people who inject drugs had pretraining participant interviews with data on injecting frequency and were included in the final analysis (mean [SD] number of interviews over the study period, 6.2 [2.2]). In fixed-effects regression analyses adjusted for covariates, there was no change in the frequency of injecting (IRR, 0.91; 95% CI, 0.69-1.20; *P* = .51), opioid injecting (IRR, 0.95; 95% CI, 0.74-1.23; *P* = .71), benzodiazepine use (IRR, 0.96; 95% CI, 0.69-1.33; *P* = .80), or the proportion of reported time of using drugs alone (IRR, 1.04; 95% CI, 0.86-1.26; *P* = .67) before and after THN training.

**Conclusions and Relevance:**

This cohort study of people who inject drugs found no evidence of an increase in injecting frequency, along with other markers of overdose risk, after THN training and supply. The findings suggest that THN training should not be withheld because of concerns about risk compensation and that advocacy for availability and uptake of THN is required to address unprecedented opioid-associated mortality.

## Key Points

**Question:**

Do people who inject drugs increase their injecting frequency (a key marker of overdose risk) after take-home naloxone (THN) training and supply?

**Findings:**

In this cohort study of 1328 people who inject drugs, no change in injecting frequency was observed after THN training.

**Meaning:**

Findings of this study suggest that THN training was not associated with increased injecting frequency and should not be withheld due to concerns about overdose risk compensation and that advocacy for widespread availability and uptake of THN is needed to address unprecedented opioid-associated mortality.

## Teaser

This cohort study examines overdose risk behaviors and take-home naloxone access among people who inject drugs participating in the Melbourne Injecting Drug User Cohort Study in Victoria, Australia.

## Introduction

Naloxone is a lifesaving antidote to opioid overdose.^1^ Take-home naloxone (THN) programs, which involve overdose-response education and naloxone supply, were developed to increase the availability of naloxone in the community among people who may be present in the event of an overdose (eg, family, friends, and companions of people who use opioids).^2^ The distribution of THN in the community has been associated with subsequent reductions in overdose deaths at the population level.^3,4^ Yet a barrier to implementation of THN programs is the perception that THN training will lead to risk compensation in individuals, potentially increasing risky drug use behaviors.^5,6,7,8,9,10,11^

Risk compensation refers to greater risk-taking due to an individual’s perception that the risk associated with an activity is diminished when public health interventions, such as THN programs, are introduced.^12,13^ Risk compensation has been discussed in the context of HIV transmission,^14,15,16^ with concerns that initiatives such as needle and syringe programs and HIV PrEP (preexposure prophylaxis) may increase sexual or injecting risk behaviors and negate the harm-reduction benefits of these initiatives.^15,17^ However, withholding lifesaving prevention strategies, because of uncertainty or a misconception that risk-taking behavior may increase, is highly contentious.^12,13^

High drug potency or dose, drug use frequency, concomitant central nervous system depressant use, and using drugs alone are known risk factors for subsequent opioid overdose.^18,19,20,21,22^ A recent systematic review by Tse et al^4^ identified a small number of studies that examined changes in heroin use as a marker of risky drug use after THN provision. Of the 5 studies included, none observed compensatory risk behavior from heroin use measures (primarily frequency of heroin use indexed as days of use), whereas some studies identified modest decreases in heroin use frequency.^23,24,25,26,27^ However, few studies that were identified in the systematic review controlled for potential confounding variables.^4^

Compared with other routes of administration, intravenous use is associated with enhanced bioavailability and absorption of drugs as well as higher overdose risk,^28^ and overdose risk increases with injection frequency.^29,30,31,32^ Despite this finding, no studies included in the systematic review by Tse et al^4^ examined as a primary outcome the changes in injection drug use frequency after THN access. One included study described the development and implementation of a THN brief intervention and reported that the mean (SD) number of days and proportion of the sample reporting injection drug use in the month before THN access (6.8 [9.6] days and 57%) decreased marginally in the month after access (6.6 [9.6] days and 55%),^25^ although these differences were not formally analyzed.

Tse et al^4^ found no studies that formally analyzed changes in reported injecting frequency while controlling for potential confounding factors in people who inject drugs who had accessed THN. This cohort study aimed to assess whether THN training is associated with changes in overdose risk behaviors by comparing risk behaviors reported before and after THN training in a cohort of people who inject drugs. We hypothesized that if THN plays a role in overdose risk compensation, then overdose-related risk behaviors, such as injecting frequency, would increase following THN training, after controlling for potential confounding variables.

## Methods

We conducted multilevel analyses of risk behaviors among participants in The Melbourne Injecting Drug User Cohort Study (SuperMIX), an active longitudinal cohort study in Australia, before and after they participated in THN training. Data from January 1, 2011, to December 31, 2021, were included. The Alfred Ethics Committee, Victorian Department of Health Human Research Ethics Committee, and Australian Institute of Health and Welfare Ethics Committee approved this cohort study. All participants provided written informed consent. We followed the Strengthening the Reporting of Observational Studies in Epidemiology (STROBE) reporting guideline.

### The SuperMIX Study

The SuperMIX study cohort comprises people who inject drugs who are followed up annually. Recruitment occurred in 2 main waves from 2008 to 2010 and in 2017, with some additional ongoing recruitment for replacement of participants who were lost to follow-up. Convenience sampling was carried out across Melbourne (ie, Frankston, Dandenong, Geelong, and metropolitan Melbourne) in Victoria, Australia. In-depth face-to-face and telephone interviews are ongoing and have been conducted approximately every 12 months. Participants were compensated A$30 (US$20.03) for each interview as reimbursement for their time and out-of-pocket expenses.

To enroll in the cohort, participants were required to be residents of Victoria, be 18 years or older, have injected heroin or methamphetamines at least 6 times in the previous 6 months, provide a valid Medicare (Australia’s universal health care system) number, and be able to give informed consent. At the first recruitment wave (conducted between 2008 and 2010), participants had to be between 18 and 30 years of age, but this criterion was relaxed over time, as was a requirement of not being in drug treatment. The full details of the SuperMIX study are provided elsewhere.^33^

### Participants

In 2017, questions regarding a history of THN training were included in the baseline and follow-up surveys of the SuperMIX study. Only participants who reported receiving THN training were included in the current analysis. For consistency across all covariates included in the analysis, interviews before June 2011 (the date that all questions contributing to the confounder variables were included in the survey) were excluded.

**Figure 1.**
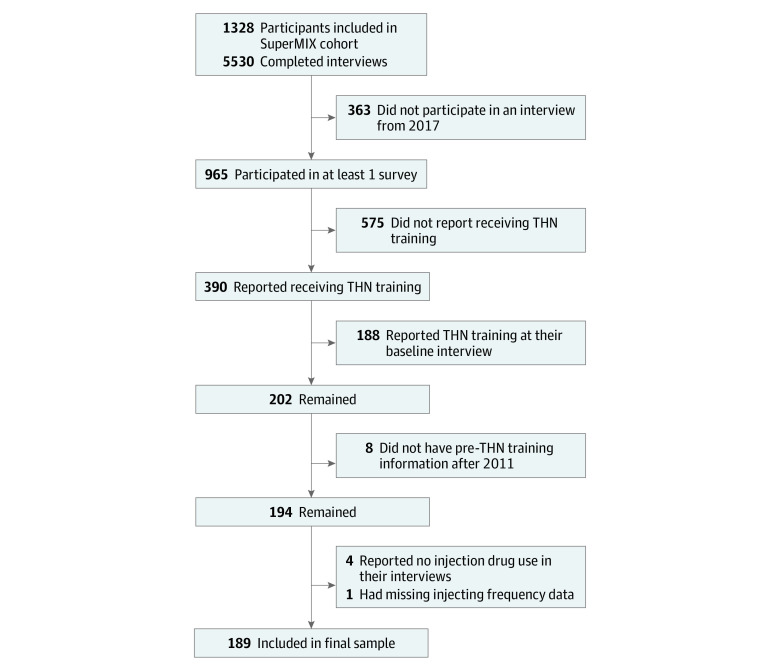
Flowchart of Participant Inclusion SuperMIX is The Melbourne Injecting Drug User Cohort Study. THN indicates take-home naloxone.

There were 1328 participants interviewed at least once between April 2008 and March 2021 and 965 participants who participated in at least 1 survey (baseline or follow-up) from 2017 onward. Of these 965 participants, 390 reported receiving THN training, but 188 reported THN training at baseline and were therefore excluded from this analysis. Of the remaining 202 participants, 8 had pretraining interviews from 2011 or earlier, 4 did not report injection drug use at any included interview waves, and 1 had missing injecting frequency data, leaving a final sample size of 189 participants who contributed a total of 933 participant interviews (Figure 1). These participants did not appear to differ from the 201 excluded participants on the outcome variables (eTable 1 in Supplement 1). They reported being older, being male, earning more money, and consuming slightly less opioids than the remaining participants; however, they did not differ on housing stability, recent overdose, or recent drug treatment.

### Measures

#### Primary and Secondary Outcomes

Injecting frequency was the primary outcome of interest. This outcome was measured by the reported total number of injections that occurred in the 7 days prior to the interview. Consistent with previous work investigating injecting frequency in this cohort,^34^ participants were asked the number of times they had injected 16 different drug types (eTable 2 in Supplement 1). Injecting frequency from every interview until the reporting of first THN training was included in the analysis (eFigure in Supplement 1).

Other overdose risk behaviors, including opioid-injecting frequency, using drugs alone, and benzodiazepine use frequency, were secondary outcomes. Participants were asked the proportion of time that they were alone when using drugs in the previous month. This measure was treated as a continuous variable that ranged from never (0% of the time) to always (100% of the time). Frequency of benzodiazepine use was also a secondary outcome and measured as the number of times benzodiazepines were used in the previous week.

#### Exposure

First THN training in the past 12 months was the exposure variable. The THN training sessions have been conducted in some alcohol and other drug programs in Victoria from 2013,^35^ with substantial expansion beginning in 2017.^36^ As part of the ongoing training, participants are instructed in overdose prevention and response and then given either intramuscular or intranasal naloxone as THN. Standardized training was offered across the state by Harm Reduction Victoria, alongside other harm-reduction services, alcohol and other drug services (including treatment services), community health centers, general practitioners (GPs), and pharmacies. We assumed that training was relatively consistent across these services, with the exception of pharmacists and GPs who were not required to undertake any of the practitioner training on offer but had access to training materials with content that was consistent with those delivered by Harm Reduction Victoria.

At interviews conducted in 2017 or later, participants were asked whether they had a history of THN training and to indicate the approximate date of that training session. In cases of participants reporting training that was more than 12 months before the interview date, the interview after the training date was included as the exposure date; for example, if the participant reported receiving THN training in February 2016 at their follow-up interview in March 2018, the participant was coded as being exposed to THN training on their March 2016 annual participant-interview observation record, with subsequent participant-interview observations excluded from the analyses. Ten participants reported THN training dates that exhibited logical inconsistency (eg, the training date was after the interview date). In this instance, their training date was assumed to be in the previous 12 months. Data from all participant interviews prior to the reported THN training were included, and data from only the first participant interview after THN training were included (eFigure in Supplement 1). Survey data after the first reported THN training session were excluded due to the assumption that engagement in risk behavior would occur immediately after training, while naloxone was on hand (ie, before being used or expiring).

#### Covariates

Time-varying factors theoretically associated with changes in injecting frequency and THN training were included in analyses. Sociodemographic factors included housing status (stable: owner occupied, renting, public housing, or shared housing; unstable: sleeping rough, living in a caravan, boarding, living rent-free with friends, house sitting, squatting, or supported accommodation) and mean weekly income (<A$250 [<US$166.61], A$250-$599 [US$166.61-US$399.20], or ≥A$600 [≥US$399.86]). Other factors were opioid overdose in the previous year, any drug treatment in the previous year (including opioid agonist treatment, rehabilitation, detoxification, or other forms of drug treatment available in Victoria), needle and syringe coverage (<100% or 100%; eMethods in Supplement 1 provides the formula for calculation),^34^ calendar year (2012-2014, 2015-2017, or 2018-2021), and time (years) in the study.

### Statistical Analysis

Given the dependencies in the data and to implicitly control for all measured and unmeasured time-invariant factors that may confound the key association of interest, we undertook fixed-effects generalized linear (Poisson) multilevel modeling using longitudinal participant-interview data to estimate the association between THN training and the primary and secondary outcomes. Time in the study was modeled as a quantitative linear function. Participant-interview data for the first reported THN training session was treated as a posttraining participant-interview record, whereas all participant-interview records before the posttraining participant interviews were considered as pretraining participant-interview records. When participants reported more than 1 THN training date, the participant interview corresponding with the earliest THN training date was treated as the exposure record. Estimates were expressed as incidence rate ratios (IRRs) with 95% CIs, and *P* values (2-tailed α = .05) were calculated to provide inference. Bootstrapped SEs (n = 1000) were estimated to provide appropriate inference in spite of possible outcome-response overdispersion. This fixed-effects estimation provides unbiased estimates, assuming the missing data process is missing at random, which indicates that missing data in the outcome can depend on the model covariates; for fixed-effects analyses, the missing data include any unobserved time-invariant factor that is not modeled.

In secondary analyses, we used generalized linear multilevel modeling to estimate the association between THN training and opioid injecting frequency, benzodiazepine use frequency, and proportion of time using drugs alone. In a sensitivity analysis, only the record immediately preceding THN training was retained as opposed to including all interviews leading up to training (ie, 2 interviews per participant).

All statistical analyses were conducted using Stata, version 16.1 (StataCorp LLC).^37^ Two-sided *P* < .05 indicated statistical significance.

## Results

### Participant Characteristics

There were 1328 participants (mean [SD] age, 32.4 [9.0] years; 893 men [67.2%], 431 women [32.5%], and 4 nonbinary or other gender identity [0.3%]) in the SuperMIX cohort at baseline (eTable 3 in Supplement 1). A total of 965 participants completed at least 1 survey from 2017 onward. Compared with participants who participated in an interview in 2017 or later but did not report receiving THN training (n = 575 [59.6%]), those who reported training at any interview (n = 390 [40.4%]) were slightly older (35.3 [9.7] vs 33.8 [9.3] years; *P* = .01), had higher mean (SD) injecting frequency (14.9 [23.7] vs 10.5 [15.3] times in the past week; *P* = .001), were more likely to report any drug treatment in the previous 12 months (56.2% vs 49.7%; *P* = .05), and were more likely to report polydrug use in the past month (82.3% vs 68.2%; *P* < .001) at baseline interview (eTable 3 in Supplement 1).

A total of 190 participants had pretraining participant-interview data on injecting frequency; however, 1 participant had missing injecting frequency data. Therefore, 189 participants were included in the final analysis (Figure 1), nearly all of whom reported using opioids at baseline (183 participants [96.8%]). Over the study period, 129 participants (68.3%) reported an opioid overdose in the previous 12 months (or since last interview) and 170 participants (89.9%) reported entering any type of drug treatment. There was a mean (SD) number of 6.2 (2.2) interviews per participant in the model (mean [SD] follow-up time, 6.5 [3.8] years).

**Figure 2.**
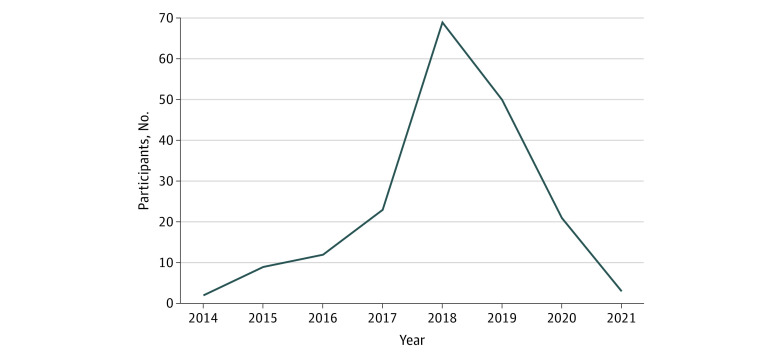
First-Reported Take-Home Naloxone Training Year

Most participants reported their first THN training session in 2018 or 2019 (Figure 2), and more than 90% of training sessions were provided by either Harm Reduction Victoria (55 [29.1%]); an alcohol and other drug service or outreach worker, including peer workers (101 [53.4%]); or a community health center (12 [6.3%]). Only 10 training sessions (11 [5.8%) were provided by a pharmacist, GP, or hospital. Two training sessions were provided in prison, and 2 training sessions were provided by housing services.

A total of 933 participant-interview observations from 189 participants were included in the analysis. Mean (SD) injecting frequency at interviews was 10.0 (14.8) times per week before training (n = 744) and 12.9 (17.3) times per week after training (n = 189). Mean (SD) opioid injecting frequency was 7.5 (10.5) times per week at pretraining interviews and 9.3 (14.1) times at posttraining interviews. Benzodiazepines were reportedly used a mean (SD) number of 4.3 (8.4) times per week before training and 3.8 (6.4) times per week after training. At 367 interviews (39.3%), participants reported either not injecting or not injecting alone. At 251 interviews (26.9%), participants reported injecting alone at either half or less than half of the time. At 315 interviews (33.8%), participants reported injecting alone more than half of the time in the past month. At pretraining interviews, the mean (SD) proportion of time that participants injected alone was 37.9% (0.4%) compared with 38.5% (0.4%) at posttraining interviews.

### Generalized Linear Multilevel Modeling and Sensitivity Analyses

After adjusting for potential confounders, there was a modest reduction in injecting frequency that was not statistically significant (IRR, 0.91; 95% CI, 0.69-1.20; *P* = .51) (Table 1). Similarly, there was no association between THN training and opioid-injecting frequency (IRR, 0.95; 95% CI, 0.74-1.23; *P* = .71), using opioids alone (IRR, 1.04; 95% CI, 0.86-1.26; *P* = .67), or benzodiazepine use frequency (IRR, 0.96; 95% CI, 0.69-1.33; *P* = .80).

**Table 1.** Unadjusted and Adjusted Fixed-Effects Poisson Regression Analysis Modeling the Association Between Take-Home Naloxone Training and Opioid Overdose Risk Behaviors^a^

Outcome	No. of interviews	IRR (95% CI)		*P* value
		Unadjusted	Adjusted^b^	
Overall injecting frequency^c^	933	1.04 (0.86-1.26)	0.91 (0.69-1.20)	.51
Opioid injecting frequency^c^	901	1.03 (0.86-1.23)	0.95 (0.74-1.23)	.71
Using drugs alone^d^	870	1.04 (0.89-1.20)	1.04 (0.86-1.26)	.67
Benzodiazepine use frequency^c^^,^^e^	753	0.92 (0.71-1.20)	0.96 (0.69-1.33)	.80

Abbreviation: IRR, incidence rate ratio.

^a^ In a fixed-effects analysis, participants who reported no change in the outcome (or exposure) over the study period did not contribute to the estimation of effect size.

^b^ Adjusted for income, housing status, time in study, recent overdose, recent drug treatment, calendar grouping, and needle and syringe program coverage.

^c^ In the previous week.

^d^ In the previous month.

^e^ There were 3 data points missing for benzodiazepine use.

We undertook the regression analyses excluding all participant-interview observations that were not immediately before training, leaving 378 participant-interview observations (189 pretraining and posttraining). For each outcome, there was no association with THN training (Table 2).

**Table 2.** Sensitivity Analysis: Adjusted Fixed-Effects Poisson Regression Analysis Modeling the Association Between Take-Home Naloxone Training and Opioid Overdose Risk Behaviors^a^

Outcome	No. of interviews	Adjusted IRR (95% CI)^b^	*P* value
Overall injecting frequency^c^	354	0.99 (0.63-1.56)	.97
Opioid-injecting frequency^c^	328	0.98 (0.63-1.54)	.94
Using drugs alone^d^	290	0.96 (0.67-1.40)	.86
Benzodiazepine use frequency^c^^,^^e^	230	1.13 (0.64-1.98)	.67

Abbreviation: IRR, incidence rate ratio.

^a^ In a fixed-effects analysis, participants who reported no change in the outcome (or exposure) over the study period did not contribute to the estimation of effect size.

^b^ Adjusted for income, housing status, time in study, recent overdose, recent drug treatment, calendar grouping, and needle and syringe program coverage.

^c^ In the previous week.

^d^ In the previous month.

^e^ There were 3 data points missing for benzodiazepine use.

## Discussion

We did not find evidence that THN training was associated with risk compensation behavior in this cohort of people who inject drugs. Rather, there was no significant change in frequency of injecting any drugs, injecting opioids, or using benzodiazepines after accessing THN. There was also no change in the proportion of time that participants reported using drugs alone, a key indicator of overdose mortality risk.^30,38,39^

There was no evidence of THN-associated compensatory risk behavior in this cohort. While not all overdose risk behaviors were examined in this study (eg, injecting in public, concomitant use of alcohol or benzodiazepines, and use of fentanyl),^40,41^ frequency of opioid injecting and frequency of benzodiazepine use are 2 of the most important risk factors for overdose. The association between knowledge of and engagement in overdose risk behaviors is complex,^38^ and THN is designed to be used on other people who may be at risk of overdose; therefore, it may be pertinent to examine the implications of naloxone availability for drug use in peer networks. In a qualitative study, participants with opioid use disorder residing in residential drug treatment programs in the US described both no change to their drug use and some engagement in riskier behavior by themselves or peers (eg, injecting heroin laced with fentanyl).^42^ However, this finding has not been borne out in empirical evidence and does not appear to correspond with increases in overdose at the population level.^3,4^

Findings from this work are consistent with an emerging evidence base suggesting that concerns about risk compensation with naloxone availability are unfounded.^4^ Yet, these concerns continue to be raised as objections for expanding THN supply.^7,11^ For example, a number of pharmacists in a recent Australian study expressed concerns about distributing naloxone, as they believed that recipients would feel comfortable increasing their opioid use.^43^ However, because naloxone administration can be associated with opioid withdrawal and reverses the effects of any opioids that have been recently taken, the outcomes of naloxone are considered unpleasant by people who inject drugs, meaning that they are typically reluctant to administer the drug.^42,44^ Furthermore, it is questionable whether this concern is reason enough to withhold a lifesaving medication from people. Only 40.4% of participants in the SuperMIX study reported THN training, despite most of the sample reporting the use of opioids. There is a clear need for widespread education among health care practitioners and other key stakeholders to enable them to address this common assumption about THN, which can act as a barrier to THN supply so that coverage is increased.

### Limitations

This study has several limitations. First, we did not analyze data on the potency or quantity of drugs that were being injected; therefore, it is possible that while frequency of injecting did not change, the potency of each injection increased; however, injecting frequency is a good proxy measure for quantity of drug use and is associated with overdose risk.^29,30,31,32^ Furthermore, previous evidence suggests that the quantity of heroin use does not change, and in some cases decreases, after THN training.^4^ Second, THN training is not necessarily the only source of THN access. Participants may have accessed naloxone through other means (eg, over-the-counter at a pharmacy, through a harm-reduction service without training, or from peers) that have not been captured in the SuperMIX data set. However, restrictions exist such that training is required by law for THN distribution. Third, THN training was targeted at people who may be present at an overdose to administer naloxone, and while most people who engage in training use opioids themselves, the sample in this cohort study may be biased to include people who are more actively engaged in harm-reduction strategies. Additionally, the findings may not be generalizable to all samples of people who inject drugs.

Fourth, as this was a prospective cohort study, participants who were included in the final subsample may not reflect all participants who reported THN training. There may be distinct differences between these 2 groups and other groups of people who inject drugs receiving THN training that limit the generalizability of the findings. Nevertheless, this study, compared with most previously published studies on this topic,^4^ used a robust repeated-measures design and a longitudinal statistical model, which, although is more prone to estimations with larger SEs, has the advantage of being able to implicitly control for any measured or unmeasured time-invariant or historical factors that might confound the associations between THN training and opioid overdose risk behavior. The longitudinal model also included adjustments for a comprehensive set of time-dependent factors (including the calendar period of interviews) that might be associated with THN training and each outcome and therefore might bias the estimated effect size of THN training in this study. Given that this study did not have a randomized design, there may still be time-dependent factors that were either not measured or omitted from analyses that may confound the associations we presented.

## Conclusions

This cohort study found no change in major overdose risk behaviors after THN training among a prospective cohort of people who inject drugs. This training should not be withheld because of concerns about risk compensation. To address unprecedented opioid-associated mortality, continued advocacy for the widespread availability and uptake of THN in the community is required.

## Notes

Supplement 1. **eTable 1.** Baseline Socio-Demographic and Drug Use Characteristics Comparing People Who Reported THN Training Before and After Their Baseline Interview **eTable 2.** Drug Types Used by Participants **eFigure.** Study Timeline With Example Participant Interview Timeline **eMethods.** Calculating Needle-Syringe Coverage **eTable 3.** Baseline Socio-Demographic and Drug Use Characteristics of the Total Cohort and People Who Reported Receiving Take-Home Naloxone (THN) Training
eReference

Supplement 2.
Data Sharing Statement
