# Supplementary material for: Injection Drug Use Frequency Before and After Take-Home Naloxone Training
Source: JAMA Netw Open. 2023 Aug 4;6(8):e2327319. doi: 10.1001/jamanetworkopen.2023.27319 (PMC10403778; doi:10.1001/jamanetworkopen.2023.27319)
Supplement: Supplement 1. — eTable 1. Baseline Socio-Demographic and Drug Use Characteristics Comparing People Who Reported THN Training Before and After Their Baseline Interview eTable 2. Drug Types Used by Participants eFigure. Study Timeline With Example Participant Interview Timeline eMethods. Calculating Needle-Syringe Coverage eTable 3. Baseline Socio-Demographic and Drug Use Characteristics of the Total Cohort and People Who Reported Receiving Take-Home Naloxone (THN) Training eReference [file jamanetwopen-e2327319-s001.pdf]

Supplemental Online Content

Colledge-Frisby S, Rathnayake K, Nielsen S, et al. Injection drug use frequency before and after take-home naloxone training. *JAMA Netw Open*. 2023;6(8):e2327319. doi:10.1001/jamanetworkopen.2023.27319

eTable 1. Baseline Socio-Demographic and Drug Use Characteristics Comparing People Who Reported THN Training Before and After Their Baseline Interview

eTable 2. Drug Types Used by Participants

eFigure. Study Timeline With Example Participant Interview Timeline

eMethods. Calculating Needle-Syringe Coverage

eTable 3. Baseline Socio-Demographic and Drug Use Characteristics of the Total Cohort and People Who Reported Receiving Take-Home Naloxone (THN) Training

eReference

This supplemental material has been provided by the authors to give readers additional information about their work.

© 2023 Colledge-Frisby S et al. *JAMA Network Open.*

eTable 1. Baseline socio-demographic and drug use characteristics comparing people who reported THN training before and after their baseline interview

Variable	Reported THN training before baseline N=201		Reported THN training after baseline N=189		Comparison	
	Mean	SD	Mean	SD	t-statistic	p-value
**Overall injecting frequency[a]**	16.4	28.1	13.2	17.7	-1.3	0.188
**Opioid injecting frequency[a]**	12.7	23.0	9.6	10.8	-1.7	0.096
**Using alone (proportion of time)**	0.41	0.4	0.40	0.4	-0.3	0.791
**Benzodiazepine use frequency[a]**	3.6	7.1	4.4	8.8	1.0	0.315
	n	%	n	%	Pearson chi^2	p-value
**Age category**					71.8	0.000
18-30	44	21.9%	102	54.0%		
30-40	62	30.8%	67	35.4%		
40+	95	47.3%	20	10.6%		
**Gender identity**					8.9	0.012
Men	142	70.6%	110	58.5%		
Women	57	28.4%	78	41.5%		
Other/Non-binary	2	1.0%	0	0.0%		
**Housing**					0.7	0.402
Stable	144	71.6%	141	75.4%		
Unstable	57	28.4%	46	24.6%		
**Income**					10.6	0.005
<$250	33	16.4%	57	30.3%		
$250-$600	136	67.7%	106	56.4%		
>$600	32	15.9%	25	13.3%		
**Drug type used (past month)**					17.4	0.001
Opioids only[b]	13	6.9%	34	18.0%		
Polydrug use (inc opioids)	172	91.5%	149	78.8%		
Non-opioids only	16	8.5%	5	2.6%		
No drugs[b]	0	0.0%	1	0.5%		
**Opioid overdose**					2.5	0.117
Past 12 months	118	62.8%	96	47.5%		
**Drug treatment**					1.0	0.320
Past 12 months	108	57.4%	111	55.0%		

[a] In the past week; [b] Excluding cannabis, tobacco, and alcohol; Among the final sub-sample, 1 participant had missing gender identity information, 2 participants had missing housing stability information, and 1 participant had missing income information at baseline.

© 2023 Colledge-Frisby S et al. *JAMA Network Open.*

eTable 2. Drug types used by participants

Drug class	Drug type
Opioids	Heroin
Opioids	Methadone
Opioids	Buprenorphine
Opioids	Morphine
Opioids	Oxycodone
Stimulants	Crystalline methamphetamine
Stimulants	Powder methamphetamine
Stimulants	Base-form methamphetamine
Stimulants	Cocaine
Stimulants	Ecstasy
Sedatives	Benzodiazepines
Selective serotonin reuptake inhibitors	Anti-depressants
Neuroleptics	Anti-psychotics
Antihistamine	Unisom gel capsules
Antiepileptics	Lyrica
-	Other types of drugs

© 2023 Colledge-Frisby S et al. *JAMA Network Open.*

eFigure. Study timeline with example participant interview timeline

In the example provided, Participant X was interviewed in December of each year they participated. In 2018, they reported THN training in September of 2017. As they were interviewed in December 2017, their subsequent participant-interviews were excluded and this interview (December 2017) was considered their final/post-THN training interview.

If the participant had reported THN training in September of 2016, and were interviewed in December 2016, 2017 and 2018, only their December 2016 participant-interview would be included and all subsequent interviews would have been excluded.

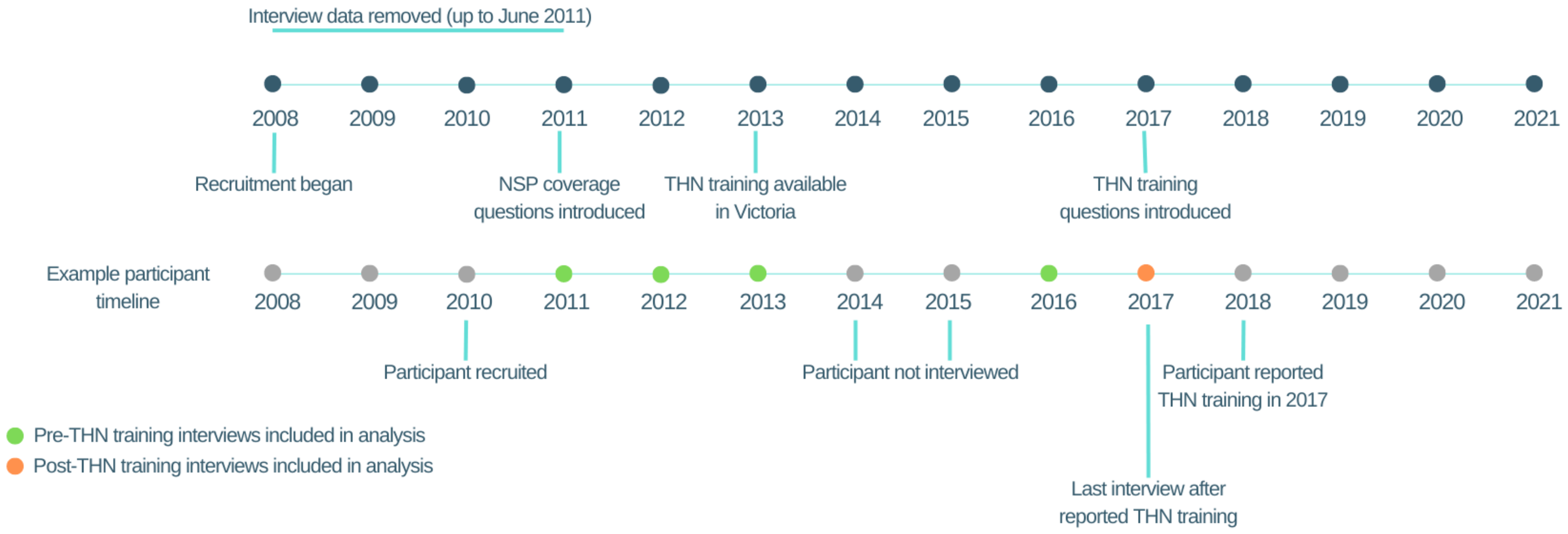

Figure note. NSP: needle-syringe program; THN: take-home naloxone.

© 2023 Colledge-Frisby S et al. *JAMA Network Open.*

eMethods. Calculating needle-syringe coverage

Consistent with previous work[1], needle-syringe coverage was measured using three variables: injecting frequency, needle-syringe acquisition, and needle-syringe distribution:

$$\text{Needle-syringe coverage} = \frac{(Needle\text{−}syringe\ acquisition) - (needle\text{−}syringe\ distribution)}{(Past\ week\ injecting\ frequency)\ x\ 2}$$

© 2023 Colledge-Frisby S et al. *JAMA Network Open.*

eTable 3. Baseline socio-demographic and drug use characteristics of the total cohort and people who reported receiving take-home naloxone (THN) training

Variable	Total cohort N=1328		Did not report THN training N=575		Reported THN training N=390		Comparison between participants who did and did not report THN training	
	Mean	SD	Mean	SD	Mean	SD	t-test	p-value
**Overall injecting frequency[a]**	11.4	18.0	10.5	15.3	14.9	23.7	3.5	0.001
**Opioid injecting frequency[a]**	8.7	14.4	7.9	13.1	11.2	18.2	3.2	0.001
**Using alone (proportion of time)**	43.8	0.4	41.2	0.4	40.3	0.4	-0.3	0.75
**Benzodiazepine use frequency[a]**	3.4	6.9	2.9	5.9	4.0	8.0	2.4	0.02
**Age**	32.4	9.0	33.8	9.3	35.3	9.7	2.5	0.01
	N	%	N	%	N	%	Pearson chi^2	p-value
**Age category**							7.4	0.03
18-30	727	54.7	266	46.3	146	37.4		
30-40	340	25.6	165	28.7	129	33.1		
40+	261	19.7	144	25.0	115	29.5		
**Gender identity**							1.9	0.59
Men	893	67.2	383	66.6	252	64.6		
Women	431	32.5	190	33.0	136	34.9		
Other/Non-binary	4	0.3	2	0.3	2	0.5		
**Housing[b]**							0.3	0.60
Stable	980	73.8	410	71.9	285	73.1		
Unstable	337	25.4	160	28.1	103	26.4		
**Income[c]**							4.8	0.09
<$250	444	33.4	161	28.3	90	23.1		
$250-$600	734	55.3	346	60.7	242	62.1		
>$600	144	10.8	63	11.1	57	14.6		
**Drug type used (past month)**							29.5	0.0000
Opioids only[b]	223	16.8	92	16.0	47	12.1		
Polydrug use (inc opioids)	965	72.7	392	68.2	321	82.3		
Non-opioids only	131	9.9	85	14.8	21	5.4		
No drugs[b]	9	0.7	6	1.0	1	0.3		
**Opioid overdose**							2.1	0.14
Past 12 months	636	47.9	288	50.1	214	54.9		
**Drug treatment**							3.8	0.05
Past 12 months	677	51.0	286	49.7	219	56.2		

[a] In the past week; [b] Excluding cannabis, tobacco, and alcohol. Note. Only participants who participated in at least one interview from 2017 onward were included in the group who did not report THN training (N=575; columns 4 and 5).

© 2023 Colledge-Frisby S et al. *JAMA Network Open.*

eReference

1. O'Keefe D, Scott N, Aitken C, Dietze P. Individual-level needle and syringe coverage in Melbourne, Australia: a longitudinal, descriptive analysis. *BMC Health Services Research* 2016; **16**(1).

© 2023 Colledge-Frisby S et al. *JAMA Network Open.*
