# Supplementary material for: Injection Drug Use Frequency Before and After Take-Home Naloxone Training
Source: JAMA Netw Open. 2023 Aug 4;6(8):e2327319. doi: 10.1001/jamanetworkopen.2023.27319 (PMC10403778; doi:10.1001/jamanetworkopen.2023.27319)
Supplement: Supplement 2. — Data Sharing Statement [file jamanetwopen-e2327319-s002.pdf]

Data Sharing Statement

Colledge-Frisby. Injection Drug Use Frequency Before and After Take-Home Naloxone Training. *JAMA Netw Open*. Published August 04, 2023. doi:10.1001/jamanetworkopen.2023.27319

Data
Data available: No

Additional Information
Explanation for why data not available: Not consistent with study ethics
